# Genetic Polymorphisms and Tumoral Mutational Profiles over Survival in Advanced Colorectal Cancer Patients: An Exploratory Study

**DOI:** 10.3390/curroncol31010018

**Published:** 2024-01-03

**Authors:** Juan Pablo Cayún, Leslie Carol Cerpa, Alicia Colombo, Dante Daniel Cáceres, José Luis Leal, Felipe Reyes, Carolina Gutiérrez-Cáceres, Susan Calfunao, Nelson Miguel Varela, Luis Abel Quiñones

**Affiliations:** 1 Laboratory of Chemical Carcinogenesis and Pharmacogenetics, Department of Basic-Clinical Oncology (DOBC), Faculty of Medicine, University of Chile, Santiago 8350499, Chile; jcayun@ug.uchile.cl (J.P.C.); leslie.cerpa@uchile.cl (L.C.C.); carolina.gutierrez@ug.uchile.cl (C.G.-C.); labcqfsusancalfunao@gmail.com (S.C.); 2 Latin American Network for Implementation and Validation of Clinical Pharmacogenomics Guidelines (RELIVAF-CYTED), Santiago 8350499, Chile; 3 Anatomy Pathology Service, Hospital Clínico de la Universidad de Chile, Santiago 8350499, Chile; acolombo@uchile.cl; 4 Department of Basic-Clinical Oncology (DOBC), Faculty of Medicine, University of Chile, Santiago 8350499, Chile; 5 Institute of Population Health, School of Public Health, Faculty of Medicine, University of Chile, Santiago 8350499, Chile; dcaceres@uchile.cl; 6 Cancer Research Department, Instituto Oncológico Fundación Arturo López Pérez, Santiago 8350499, Chile; jlleal@gmail.com (J.L.L.); freyesc@gmail.com (F.R.); 7 Department of Pharmaceutical Sciences and Technology, Faculty of Chemical and Pharmaceutical Sciences, University of Chile, Santiago 8350499, Chile; 8 Laboratory Pathological Anatomy, Hospital Luis Calvo Mackenna, Santiago 8350499, Chile

**Keywords:** colorectal cancer, pharmacogenomics, biomarkers

## Abstract

Colorectal cancer is a common disease, both in Chile and worldwide. The most widely used chemotherapy schemes are based on 5-fluorouracil (5FU) as the foundational drug (FOLFOX, CapeOX). Genetic polymorphisms have emerged as potential predictive biomarkers of response to chemotherapy, but conclusive evidence is lacking. This study aimed to investigate the role of genetic variants associated with 5FU-based chemotherapy on therapeutic response, considering their interaction with oncogene mutations (*KRAS*, *NRAS*, *PI3KCA*, *AKT1*, *BRAF*). In a retrospective cohort of 63 patients diagnosed with metastatic colorectal cancer, a multivariate analysis revealed that liver metastases, *DPYD*, *ABCB1*, and *MTHFR* polymorphisms are independent indicators of poor prognosis, irrespective of oncogene mutations. *BRAF* wild-type status and high-risk drug-metabolism polymorphisms correlated with a poor prognosis in this Chilean cohort. Additionally, findings from the genomics of drug sensitivity (GDSC) project demonstrated that cell lines with wild-type BRAF have higher IC50 values for 5-FU compared to *BRAF*-mutated cell lines. In conclusion, the genetic polymorphisms *DPYD* *rs1801265*, *ABCB1* *rs1045642*, and *MTHFR* *rs180113* may serve as useful biomarkers for predicting a poor prognosis in patients undergoing 5-fluorouracil chemotherapy, regardless of oncogene mutations.

## 1. Introduction

Colorectal cancer (CRC) is still one of the leading causes of death in Chile and worldwide, and it is defined as malignant neoplasia that develops from the colon or rectum epithelial tissue [1,2]. A higher incidence of CRC is observed in developing countries with an increasing Human Developed Index (HDI) characterized by a higher prevalence of risk factors such as obesity, low physical activity, and low socioeconomic status [3]. As of 2020, the mortality rate due to colorectal cancer in Chile was 11.0 and 8.1/100,000 inhabitants, in men and women, respectively [4]. The survival rate for colorectal cancer is variable and depends on the stage of diagnosis, among other factors. Approximately 50% to 60% of patients diagnosed with colorectal cancer develop metastases, and 80% to 90% of these patients have unresectable metastatic liver disease [2]. Colorectal cancer recurrence after curative therapy (surgery followed by adjuvant chemotherapy) occurs in 80% and 95% of cases in the first 3 and 5 years, respectively [5,6]. The median overall survival in the metastatic setting has been estimated between 15.0 and 40.3 months and depends, among other factors, on the clinical characteristics, the tumor sidedness, and some molecular characteristics (*KRAS*, *BRAF*, Microsatellite instability). which are prognostic and eventually predictive for certain systemic therapies [7].

The treatment of metastatic CRC improved significantly with the incorporation of 5-fluorouracil (5-FU) in chemotherapy regimens in combination with leucovorin (LV) [8] and remains the backbone of most systemic treatments. Capecitabine, a prodrug of 5-fluorouracil, has similar efficacy [9]. The addition of oxaliplatin (FOLFOX regimen) to 5-FU improves the response rate and progression-free survival compared to 5-fluorouracil [10,11]. Capecitabine in combination with oxaliplatin (CAPEOX) is non-inferior to FOLFOX in first-line metastatic colorectal cancer [9]. Irinotecan (CPT-11) combined with 5-FU/LV (FOLFIRI) is another option in advanced colorectal cancer, with a different toxicity profile, but is considered equivalent to FOLFOX and CapeOx [12,13]. Targeted therapies, such as EGFR inhibitors (cetuximab, panitumumab), antiangiogenic agents (bevacizumab), and BRAF/MEK inhibitors [14], have shown benefits in advanced metastatic disease, where these antibodies have an established role [1,2,14,15], whereas targeted treatment for KRASG12C mutations is in development (e.g., sotorasib (AMG 510), adagrasib (MRTX849)).

5-FU is primarily metabolized by the dihydropyridine dehydrogenase (DPD) enzyme (>80%) to 5,6-dihydro-5-FU. DPD is found primarily in liver and gastrointestinal tissue and has been identified as the main source of inter-patient variability in the pharmacokinetics of 5-FU. This variability is mainly explained by genetic polymorphisms in the *DPYD* gene, which encodes the DPD protein with different polymorphic variants: c.1905+1 G>A, c.1679T>G, c.1236G>A/HapB3, c.1601G>A, and c.2846A>T [16]. The effects of these genetic variants on DPD enzyme expression levels, affecting the pharmacokinetics process, are well documented [17,18], as are the effects on 5-FU metabolism [17,18,19]. In the *DPYD* gene, c.1679T>G and c.1236G>A/HapB3, DPYD*2A, and c.2846A>T are predictors of the toxicity generated by 5-fluorouracil regimens [13,18]. Presently, CPIC and DWPG guidelines recommend *DPYD* genotyping to mitigate toxicity in metastatic colorectal treatments [16,20,21]. Numerous studies have highlighted the advantages of *DPYD* genotyping in averting severe toxicity, demonstrating its potential cost-effectiveness compared to standard care [22,23,24]. However, despite this recommendation, the impact of these *DPYD* polymorphisms on chemotherapy efficacy remains controversial.

Similarly, mutations in *ABCs* transporter genes have been identified as significant contributors to colorectal cancer (CRC) progression and patient survival. The distribution process of 5-fluorouracil in membranes, cells, blood, and tissues depends on ABC transporters. Studies have shown that mutations in the *ABCB1* gene, encoding MDR1 (P-glycoprotein), can lead to multidrug resistance in CRC cells, resulting in a poor response to chemotherapy [25]. Gene expression analyses reveal the predictive value of low expression of *ABCB1* mRNA and poor overall survival in the TCGA cohort [26]. Additionally, alterations in the *ABCC2* gene, encoding MRP2, have been associated with unfavorable clinical outcomes and reduced overall survival in CRC patients [27,28]. These findings highlight the importance of *ABC* transporter mutations as prognostic factors and their role in therapeutic resistance in CRC. Further investigation into the mechanisms underlying these mutations and the development of targeted therapies is warranted to improve patient outcomes.

At the site of action, 5-fluorouracil undergoes conversion to fluorodeoxyuridine monophosphate (FdUMP), a molecule that impedes thymidylate synthase (TS), thereby triggering the generation of deoxythymidine monophosphate (dTMP). During this process, the folate-derived cofactor 5,10-methylenetetrahydrofolate (5,10-MTHF) operates as a methyl donor, undergoing metabolism by methylenetetrahydrofolate reductase (MTHFR). Another metabolite of 5-fluorouracil, 5-FdUMP, when combined with 5,10-MTHF, irreversibly inhibits TS. This sequence of events culminates in the disruption of DNA replication and repair mechanisms, consequently fostering cytotoxicity [29,30,31]. Therefore, genetic variations within *TYMS* (the gene encoding TS) and MTHFR have become focal points of investigation in association studies [30,31].

Oxaliplatin is utilized in conjunction with 5-fluorouracil for treating colon cancer, administered through the FOLFOX and CAPEOX regimens. The formation of oxaliplatin-DNA adducts obstructs DNA replication, ultimately leading to the demise of cancer cells. The nucleotide excision repair (NER) pathways are involved in the recognition and repair of these adducts. *ERCC1* and *ERCC2* (excision repair cross-complementation groups 1 and 2, respectively) genes have been focal points of association studies in this context [32,33]. Additionally, the elimination of oxaliplatin involves the action of glutathione S-transferase (GST), a superfamily of dimeric phase II metabolic enzymes responsible for detoxifying platinum drugs. A polymorphism in exon 5 of the *GSTP1* (glutathione S-transferase P1) gene results in an isoleucine-to-valine substitution at the 105th amino acid (Ile105Val), leading to decreased GSTP1 activity. This, in turn, affects the accumulation of oxaliplatin within cancer cells [33].

On the other hand, the tumor mutational status in colorectal cancer has been an important point of interest in finding efficacy biomarkers. In colorectal cancer, *KRAS*, *NRAS*, *BRAF*, and *PIK3CA* mutations induce a negative effect on the response to anti-EGFR therapies [34,35]. Specifically, only *KRAS*-wild-type patients are candidates for anti-EGFR treatments. In addition, BRAF-mediated signaling (*RAS-RAF-MEK-ERK-MAP* kinase pathway) is associated with poor prognosis, mainly the V600E mutation in the kinase domain of the protein that generates a conformation that leads to constitutive activation [34]. The *BRAF V600E* mutation occurs in 8.2% of mCRC and is associated with poor survival. In *BRAF V600E* patients, 21.2% have poor mismatch repair (dMMR) versus 3.6% of dMMR in *BRAF* wild-type patients. Both markers are associated with a poor response [31]. *BRAF V600E* in patients with metastatic CRC is a predictor of response to BRAF/MEK inhibitors and is a standard target [14]. In addition, *PIK3CA*, encoding the catalytic subunit of the phosphoinositide 3-kinase (PI3K) pathway, is frequently mutated in CRC and has a significant impact on patient survival. Dysregulation of the PI3K pathway due to *PIK3CA* mutations promotes tumor progression and resistance to therapy, leading to adverse patient outcomes. Various studies have reported the prevalence of *PIK3CA* mutations in CRC ranging from 10% to 20%, with hotspot mutations such as H1047R and E545K being the most common. These mutations result in constitutive activation of the PI3K pathway, leading to enhanced cell proliferation and survival [36]. Several studies have indicated that CRC patients harboring *PIK3CA* mutations have poorer overall survival compared to those without these mutations [37,38,39].

Both tumor mutational status and drug-metabolism polymorphisms have the potential to affect the prognosis of colorectal cancer patients. For example, *EGFR* mutations in exon 19 are correlated with high expression of *ERCC1* (the oxaliplatin-related gene), low expression of the *TYMS* gene, and poor prognosis in lung cancer patients [40]. Furthermore, in vitro studies in lung cancer cells showed that *EGFR* exon 19 mutations increase DPD expression through the transcriptional factor SP1 [41]. This regulation of DPD may explain the limited benefit of tegafur (5-FU prodrug) in patients with *EGFR* exon 19 mutations because tegafur delivers 5-FU with subsequent metabolization by DPD.

In colorectal cancer, resistance to 5-fluorouracil chemotherapy is associated with increased expression of DPD and a possible increase in thymidylate synthase [42]. Clinical studies have shown that 5-FU and oxaliplatin-based regimens in metastatic colorectal cancer increase ERCC1 mRNA, thymidylate synthase, and DPD, and this effect is associated with decreased survival [43,44]. Recent evidence underscores the predictive value of ERCC1 mRNA in gauging chemotherapy efficacy [45].

The sole study in colorectal cancer that established an association between *KRAS* mutation and *DPYD* variations demonstrated that the -c.496A>C *DPYD* variant is exclusively present in patients with wild-type *KRAS* [46]. The potential interplay or distinct impact of mutations within the EGFR pathway (*EGFR*, *KRAS*, *NRAS*, *BRAF*, *PI3KCA*), along with polymorphisms in genes related to 5-fluorouracil and oxaliplatin (*DPYD*, *TYMS*, *GSTP1*, *MTHFR*, *ABCB1*), on the prognosis of colorectal cancer remains uncharted territory.

Hence, the primary goal of this study is to elucidate the potential influence of *EGFR* mutations and drug-gene polymorphisms on overall survival within a Chilean cohort. Furthermore, the TCGA COARED cohort was employed to elucidate gene expression patterns in the interplay between germline polymorphisms and oncogene mutations. Additionally, the Genomic Cancer Drug Sensitivity Database was utilized to observe how mutational status impacts sensitivity to 5-fluorouracil.

## 2. Materials and Methods

### 2.1. Patients and Tissue Sampling

Formalin-fixed paraffin-embedded (FFPE) CRC samples (63 sixty-three) were obtained from patients at the National Cancer Institute in Chile and the Clinical Hospital at the University of Chile. The selection criteria were older than 18-year-old adults and histologically diagnosed with stage IV colorectal cancer, adenocarcinoma histology, and 5-fluorouracil-based chemotherapy (first line of treatment, FOLFOX/CapeOx). The main goal was to acquire molecular biomarkers linked to overall survival. To achieve this, a retrospective patient selection was conducted, spanning from 2016 to the recent past, in conjunction with a thorough chart review. The assessment of overall survival was tracked until April 2022 (Survival sweep). The measurements and variables are presented in the Supplementary Materials (Table S1). The primary variables utilized encompassed age, gender, localization, colectomy, liver metastases (the most prevalent), metastasectomy, and the utilization of radiotherapy, biological antibodies, and second-line treatments. This study was approved by the Ethics Committee of the North Health Service of the Metropolitan Region in accordance with Good Clinical Practice (GCP), the Declaration of Helsinki, and the International Conference of Harmonization (ICH).

The determination of the proportion of tumor and normal cells was visually carried out by a team of pathologists affiliated with the Biobank of Fluids and Tissues at the University of Chile (BTUCH). Each patient participating in this study contributed both tumor and normal samples for analysis. Tumor samples were categorized as FFPE slices exhibiting less than 10% necrosis and less than 50% non-neoplastic tissue. These samples were utilized to extract tumor DNA. Meanwhile, normal samples were identified as FFPE slices with less than 10% necrosis and less than 20% tumor tissue. These samples were collected for the purpose of obtaining germline DNA. It’s worth noting that, due to the retrospective design of this study, acquiring germline DNA from blood samples was not feasible.

In addition, the TCGA Colon Cancer cohort (Pan Cancer Atlas) was included in the analysis to compare the effect of those polymorphisms on other external samples and evaluate gene expression. The expression of *TYMS* and *DPYD* mRNA data were obtained and downloaded from cBioportal. The mRNA expression used a z-score of 2 and compared the tumor samples versus normal samples (https://www.cbioportal.org/; accessed on 30 October 2023) [46]. A total of 74 patients from the TCGA consortium were included in the analysis of DPD expression, and 106 patients from the TCGA consortium were included in the analysis of Thymidylate synthase pathway expression (TYMS, TK1, TYMP, and FOXM1) [47].

### 2.2. Molecular Testing

The extraction and purification of DNA and RNA from FFPE samples were performed using the Qiagen AllPrep DNA/RNA FFPE kit according to the manufacturer’s instructions. Briefly, fresh FFPE tissue (2–4 sections of 10–20 µm) containing > 50% tumor cells were deparaffinized and incubated in a lysis buffer containing proteinase K. The mixture was centrifuged to precipitate the DNA, leaving the RNA in the supernatant. In addition, freshly cut FFPE tissue (10–20 µm sections) containing normal cells was used for similar DNA and RNA extraction. DNA quality control was carried out using a 260 nm/280 nm ratio assessment and 2% agarose gel electrophoresis. RNA samples were preserved for future studies. The mutational profile of tumor DNA was analyzed using the EntroGen^®^ Colon Cancer mutation detection panel (CRC-RT48), specifically designed for tumor DNA. Genotyping of drug-metabolism-related genes was conducted using the *TaqMan*^®^ assay with germline DNA. The selected polymorphisms for this study were chosen based on their relevance in previous research. To validate germline polymorphism results, assays were performed on both tumor DNA and germline DNA, revealing consistent findings. Further details on the *TaqMan*^®^ assays can be found in the supplementary material.

### 2.3. Drug Sensitivity Analysis

Drug sensitivity data (bulk data) for colon and rectum adenocarcinoma cell lines (COREAD) were obtained from “The Genomics Cancer Drug Sensitivity” database (https://www.cancerrxgene.org/; accessed on 30 October 2023) [48]. COREAD classification was used to compare the mutational profile and Ln IC50 values to 5-fluorouracil. The mutational profile includes the following mutations: *EGFR*, *KRAS*, and *BRAF*. The comparison between mutated cell lines and wild-type cell lines was tested using the Wilcoxon test (non-parametric).

### 2.4. Statistical Analysis

A descriptive analysis was used to characterize the patients. Overall survival (OS) was evaluated up to 60 months of follow-up. Out of the total seventy-three (73) patients analyzed, 60 patients were eligible for inclusion in the survival analysis. Kaplan–Meier analysis with a log-rank test and multivariate Cox regression models (step-wise method) were used to evaluate the effect of mutational profiles and drug-metabolism polymorphisms on therapeutic responses. Statistical significance was determined by a *p*-value < 0.05. Given the relatively small cohort, the multivariate analysis involved testing models with 60 patients or fewer, depending on the presence of missing data in certain variables. The time from the start of the diagnosis to death from any cause was monitored to perform the survival analysis. All analyses were conducted using R Studio software (version 1.4.1717) with various data analysis packages for fundamental operations (“psych”, “summary tools”, “table1”, “dplyr”, “gtsummary”) and visualization (“ggplot2”). Additionally, survival analysis utilized packages such as “survival” and “survminer” [49].

## 3. Results

### 3.1. Patient Characteristics

**Figure 1:**
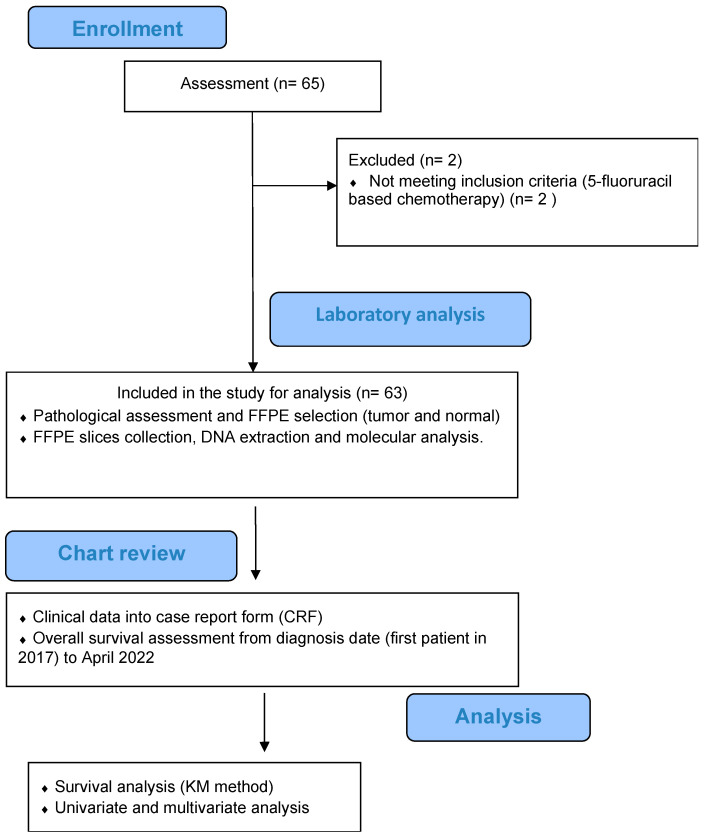
Flowchart of disposition of patients within this study.

A total of sixty-three (63) patients were finally included in this report. Demographic and pathological characteristics are presented in Table 1. Figure 1 illustrates a flowchart depicting the process of patient selection. The first-line regimen used for all patients consisted of FOLFOX/CapeOx. The median age was 66.4 years (range: 30.4–81.8), and 32 patients were females (50.8%). The primary tumor origin was left in 46 (73.0%) patients and right in 15 (17.5%) patients. Monoclonal antibody therapy (cetuximab, panitumumab, and bevacizumab) was used in 14/63 patients (22.2%). A second line of treatment was used in 37/63 patients (56.8%). Liver metastases were present in 73.0% of patients, while other types of metastases were less frequent (lung metastases 46%, other metastases 42%).

### 3.2. Molecular Profile

Table 2 shows the germline DNA variations. The genotypic frequency of *TYMS* del-del 3′UTR *rs151264360* was presented in 31 of 63 patients (49.2%). The *GSTP1 rs1695 *G/G genotype was found in 15 of 65 patients (23.8%). In the *DPYD* *rs1801265* *c.85T>C* characterization, the genotype G/A was found in 19 patients of 63 (30.2%), and the A/A genotype was found in 37 patients of 63 (58.7%). The *ABCB1* *rs1045642* *C4535T *C/C was presented in 21 patients (33.3%), the *ABCB1* *rs1128503* *C1236T *C/C was presented in 15 of 63 patients (23.8%), the *ABCC2 rs717620 *C/C was presented in 46 of 63 patients (73.0%), the *MTHFR rs1801131 *A/A was presented in 33 of 63 patients (52.4%), and the *ERCC2 rs13181 *G/G was presented in 25 of 63 patients (39.7%). The mutational profile in tumor DNA is presented in Table 3. Seven patient tumors (11.1%) had *PI3KCA* gene mutations. *KRAS* and *BRAF V600E* were detected in 22 (34.9%) and 7 (11.1%) patients, respectively.

### 3.3. Correlations of Clinicopathological Characteristics and Mutation Profile with Overall Survival

**Figure 2:**
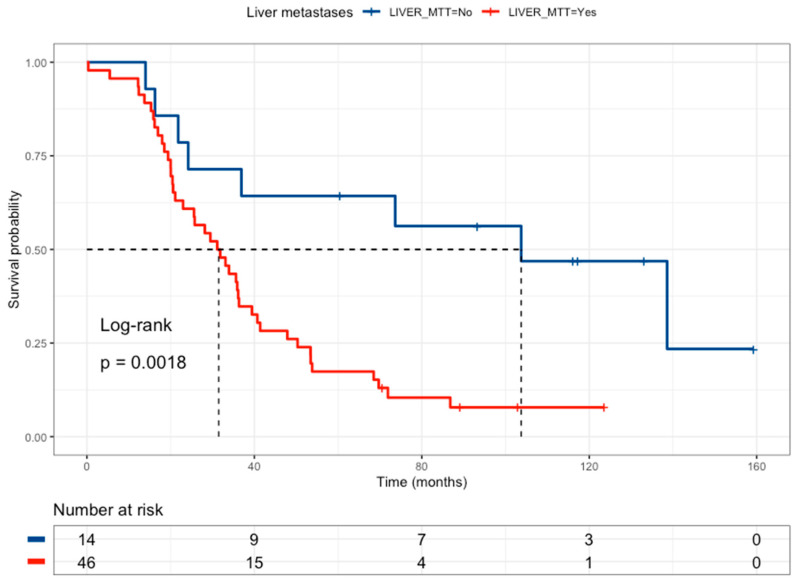
Kaplan–Meier curve of colorectal cancer patients according to liver metastasis status (without liver metastases = blue line and a median survival of 103.7 months, with liver metastases = red line and a median survival of 31.5 months).

Among the 63 patients analyzed, 53 died, with a median survival of 33.9 months and a median follow-up of 117 months. After the eligibility to association study, 60 patients were considered eligible by liver metastases, of which 50 died (42 with liver metastases and 8 without liver metastases) with a median follow-up of 102 months in the liver metastases group and 117 without liver metastases, revealing that liver metastases are related to poor survival (HR = 3.51, 95% CI 1.52–8.07) (Figure 2 and Table 4). Regarding the *BRAF V600E* mutation, 61 patients have been considered eligible for survival analysis, of which 53 died (3 mutated and 50 wild-type) with a median follow-up of 117 months for mutated patients and 133 months for wild-type patients, revealing that *BRAF V600E* wild-type status correlated with better survival than *BRAF V600E* patients (HR = 0.28, 95% CI 0.087–0.909) (Table 4). Survival analysis according to *KRAS* mutations considered 63 patients, of whom 53 died (18 KRAS-mutated patients and 35 wild-type patients), with a median follow-up of 102 months for mutated patients and 117 for wild-type patients, revealing that KRAS mutations had no association with overall survival. Additionally, survival analysis according to PI3KCA mutations considered 63 patients, of whom 53 died (3 mutated patients and 50 wild-type patients), with a median follow-up of 102 months for mutated patients and 123 for wild-type patients, revealing that the *PI3KCA* mutation correlated to better survival than *PI3KCA wild-type* (HR = 0.271, 95% CI 0.84–0.876) (Table 4). In drug-metabolism polymorphisms, the survival analysis according to *GSTP1 rs1695* considered 63 patients, of whom 53 died (10 G/G patients and 43 G/A+A/AA patients), with a median follow-up of 133 months for G/G patients and 117 months for G/A+A/A patients, indicating that the *GSTP1* *rs1695* G/G genotype was associated with a better overall survival compared with the *GSTP1 rs1695* G/A + A/A genotype, HR = 0.484 (0.234–1.00) (Table 4). Finally, survival analysis according to *DPYD rs1801265* considered 62 patients, of whom 53 died (23 T/C+C/C patients and 30 T/T patients), with a median follow-up of 102 months for T/C+C/C patients and 117 months for T/T patients, indicating that *DPYD rs1801265* T/C and C/C genotypes (HR = 1.819, 95% CI 1.03–3.19) (Table 4) correlated with poor survival. Also, the analysis of *ABCB1 rs1045642* included 61 patients, of whom 52 died with a median follow-up of 117 months in T/T+T/C patients, revealing that the *ABCB1 rs1045642* C/C genotype correlated with poor survival (HR = 1.782, 95% CI 1.03–3.19 (Table 4). Regarding MTHFR *rs180113*, 62 patients were included, of whom 53 died with a median follow-up of 89.1 months for A/C +C/C patients and 117 months for A/A patients, indicating that the *MTHFR rs180113* C/C genotype correlated with poor survival (HR = 2.295, 95% CI 1.05–4.97, Table 4). Finally, 63 patients were included in the *TYMS rs151264360* analysis (31 del/del patients and 32 ins/del patients), of whom 53 died (29 del/del patients and 24 ins/del patients), with a median follow-up of 123 months for ins/del patients, revealing that the *TYMS rs151264360* del/del genotype correlated with poor survival (HR = 2.169, 95%CI 1.21–3.86) (Table 4).

**Figure 3:**
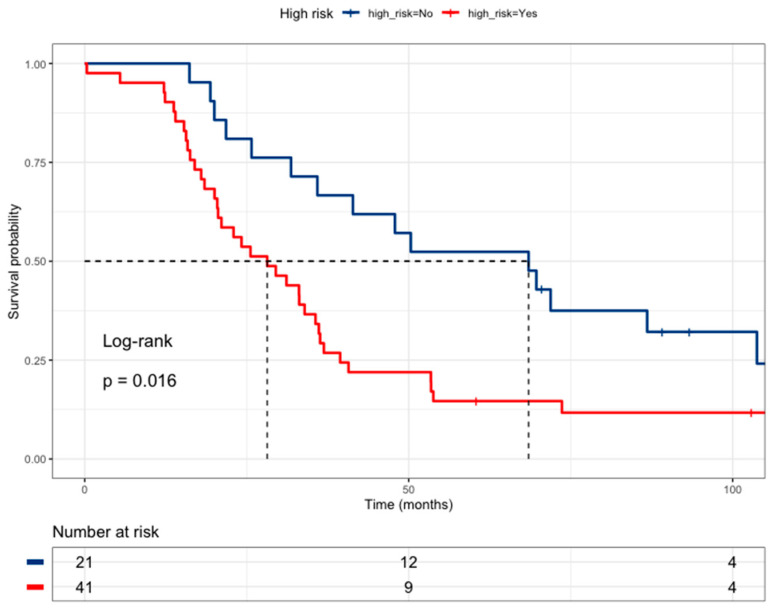
Kaplan–Meier curve of colorectal cancer patients according to High-Risk (*DPYD rs1801265* T/C + C/C genotypes, *ABCB1 rs1045642 *C/C genotype, and *MTHFR rs180113* C/C genotype) (Low risk = blue line (median survival of 68.5 months), High-risk = red line (median survival of 28.2 months)).

An initial combinatory analysis was undertaken to identify a high-risk profile among drug-metabolism polymorphisms. The high-risk profile was defined as the concurrent presence of at least one risk group from *DPYD rs1801265* T/C + C/C genotypes, *ABCB1 rs1045642 *CC genotype, and *MTHFR rs180113* C/C genotype. The presence of this high-risk profile exhibited a correlation with poorer survival (HR = 2.06, 95% CI 1.13–3.74), as illustrated in Figure 3 and outlined in Table 4. The high-risk profile is not associated with liver metastases. The selection of these genotypes was based on the multivariate Cox regression model (Table 5).

Multivariate analysis included all variables with a *p*-value < 0.1 using a step-wise procedure. The samples used for the multivariate analysis included both tumor mutations and genetic polymorphisms. Table 5 shows the multivariate final model, where liver metastasis presence (HR = 3.69, 95% CI 1.49–9.09), *DPYD rs1801265* C/C genotype (HR = 1.88, 95% CI 0.99–3.54), *ABCB1 rs1045642* C/C genotype (HR = 2.62, 95% CI 1.37–4.99), and *MTFHR rs180113* C/C genotype (HR = 2.63, 95% CI 1.13–6.15) were poor survival biomarkers (Table 5).

**Figure 4:**
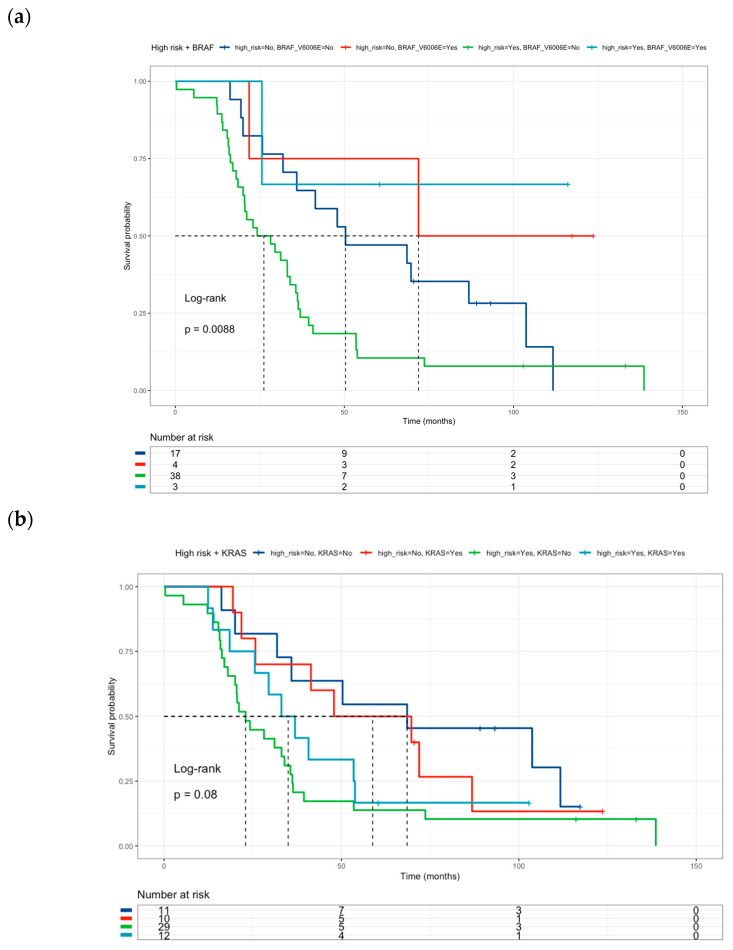

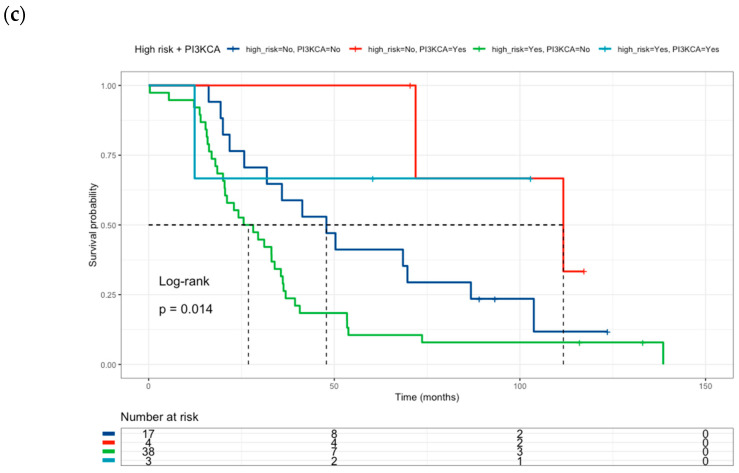
Kaplan–Meier curves of colorectal cancer patients according to: (**a**) High-Risk profile and *BRAF* mutational status (Low risk and *BRAF V600E wild-type* = blue line (median survival of 50.3 months), Low risk and *BRAF V600E mutated* = red line (median survival of 71.9 months), High-risk and *BRAF V600E wild-type* = green line (median survival of 26.2 months), High-risk and *BRAF V600E mutated* = sky blue line (median survival not reached). (**b**) High-Risk profile and *KRAS mutational status:* (Low risk and *KRAS wild-type* = blue line (median survival of 68.5 months), Low risk and *KRAS mutated* = red line (median survival of 58.8 months), High-risk and *KRAS wild-type* = green line (median survival of 23.0 months), High-risk and *KRAS mutated* = sky blue line (median survival of 35.0 months). (**c**) High-Risk profile and *PI3KCA mutational status:* (Low risk and *PI3KCA wild-type* = blue line (median survival of 47.9 months), Low risk and *PI3KCA mutated* = red line (median survival of 111.7 months), High-risk and *PI3KCA wild-type* = green line (median survival of 26.9 months), High-risk and *PI3KCA mutated* = sky blue line (median survival not reached). Risk genotype profile includes *DPYD rs1801265* T/C + C/C genotypes, *ABCB1 rs1045642 *C/C genotype, and *MTHFR rs180113* C/C genotype).

The effect of high-risk classification on drug-metabolism polymorphisms was tested together with oncogene mutation status. Neither *BRAF V600E* (Figure 4a) nor *KRAS* mutations (Figure 4b) were associated with survival in the multivariate analysis (Table 6). However, *PI3KCA* mutated status (Figure 4c) correlated with better survival than *PI3KCA* wild-type patients (HR = 0.22, 95% CI 0.05–0.95) (Table 6) in this multivariate and combinate model that considers high-risk presence and liver metastasis presence.

**Figure 5:**
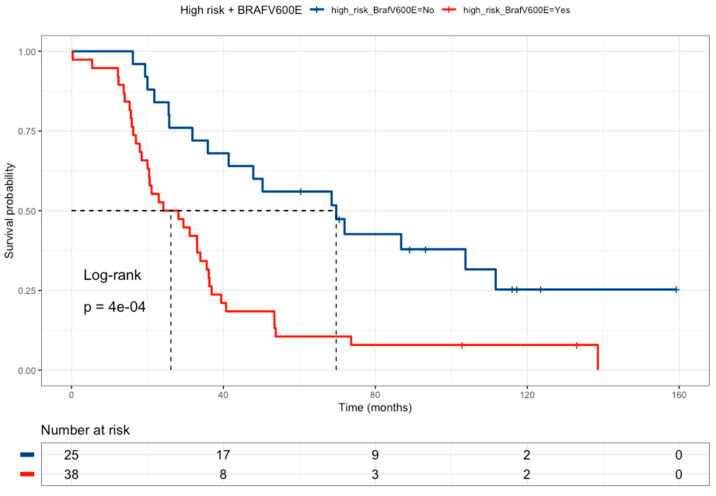
Kaplan–Meier curve of colorectal cancer patients comparing High-Risk profiles and *BRAFV600E* wild-type patients (red line, median survival of 26.2 months) versus all the other patients (blue line, median survival of 69.7 months). Patients with a High-Risk genotype profile (any of the following polymorphisms: *DPYD rs1801265* T/C + C/C *genotypes*, *ABCB1 rs1045642* C/C *genotype*, *and MTHFR rs180113* C/C *genotype*) must have the *BRAF* wild-type.

The effect of *BRAF* wild-type and high-risk drug-metabolism polymorphisms was tested as an independent group compared with all other patients (Figure 5). In this analysis, patients were grouped based on the presence of the high-risk drug-metabolism polymorphism, or BRAF-wild-type, versus the other patients. The combination of these groups correlated with a poor prognosis (HR = 2.71, 95% CI 1.46–5.01) (Table 7).

**Figure 6:**
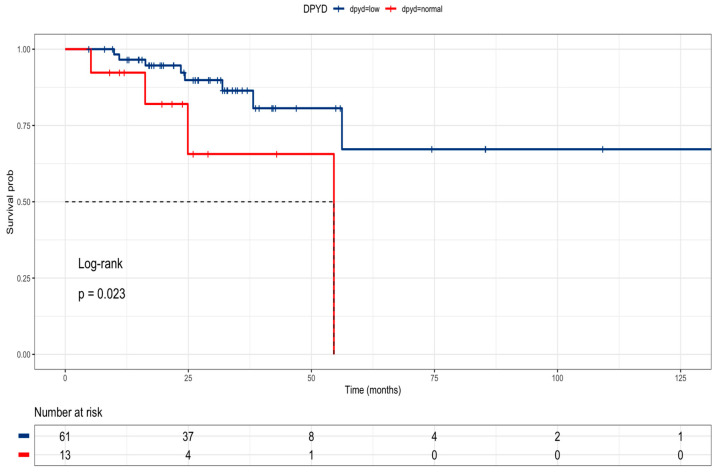
Kaplan–Meier curve of stage III colon cancer patients according to DPYD expression in the TCGA cohort (DPD low = blue line, DPD normal = red line).

**Figure 7:**
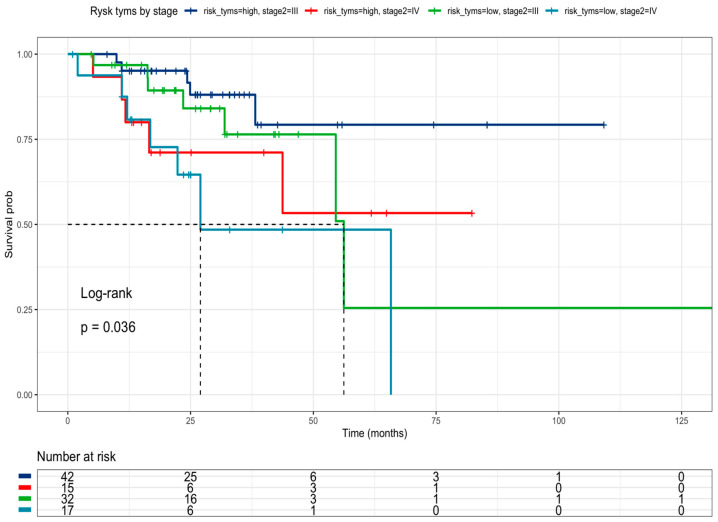
Kaplan–Meier curve of TCGA colon cancer patients according to *TYMS*, *TK1*, *TYMP*, and *FOXM1* expression in the TCGA cohort by clinical stage.

The research delved into public data from the TCGA COARED cohort to assess how the gene expression of drug metabolism genes influences tumor responses. In the COARED cohort, low expression of DPYD appeared to correlate with improved survival compared to normal DPYD expression, specifically in stage III (Figure 6). While the link between TYMS expression and outcomes didn’t reach significance, elevated expression levels of TYMS, TK1, TYMP, and FOX1 were linked to extended overall survival in both stage III and stage IV patients (Figure 7).

### 3.4. Drug Sensitivity Analysis

The interaction between drug metabolism genes and tumor responses was explored using publicly available in vitro data. The Genomic Cancer Drug Sensitivity Database was utilized to examine how cell lines derived from the colon respond to 5-fluorouracil and how this response correlates with mutational status.

**Figure 8:**
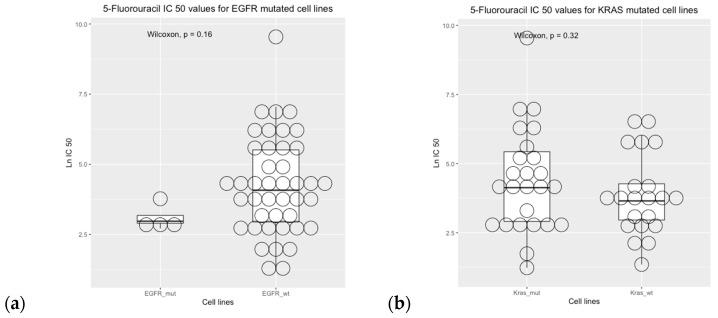

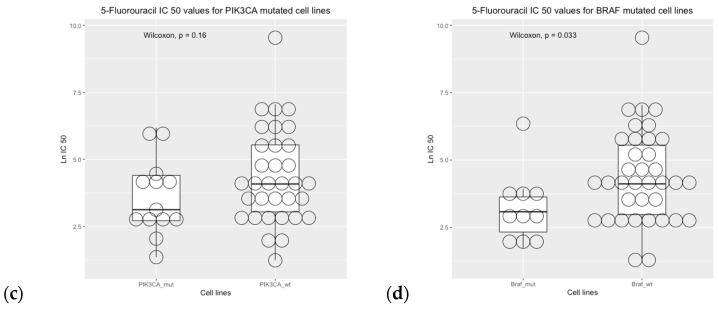
Drug sensitivity analysis of COREAD (Colon and rectum adenocarcinoma) cell lines to 5-fluorouracil (GDSC2 dataset, Sanger Screening Site, *n* = 968). Each circle represents one cell line. The data were obtained from “Genomics of Drug Sensitivity” (https://www.cancerrxgene.org/; accessed on 30 October 2023) [48].

Cell line sensitivity to 5-fluorouracil showed that IC50 was higher in *BRAF* wild-type cell lines versus *BRAF* mutated cell lines. The comparisons between mutational status and IC50 values were not statistically significant for the *EGFR*, *KRAS*, and *PIK3CA* genes (Figure 8).

## 4. Discussion

This is a retrospective study of 63 patients with CRC treated with FOLFOX/CapeOx as first-line treatment in the Chilean population. The correlation between *TYMS*, *GSTP1*, *DPYD*, and *ABCB1* gene variation and oncogene mutations (*KRAS*, *NRAS*, *BRAF*, and *PI3KCA*) is poorly understood in the literature. Here, we report the high-risk of genetic polymorphisms associated with the overall survival of colon cancer patients. The high-risk profile includes *DPYD rs1801265* T/C + CC *genotypes*, *ABCB1 rs1045642 *CC *genotype*, *and MTHFR rs180113* C/C *genotype.* Our results indicated that the *BRAF V600E* mutation was associated with better overall survival and higher sensitivity to 5-fluorouracil, obtained from publicly available data from the Genomic Cancer Drug Sensitivity Database. In addition, the presence of both *BRAF* wild-type and high-risk drug metabolism polymorphisms correlated with poor prognosis.

Furthermore, this study found that a mutated *PI3KCA* status was linked to improved survival. However, the statuses of *EGFR*, *NRAS*, and *KRAS* did not show a connection with overall survival, although these findings are constrained due to a small sample size. In both univariate and multivariate Cox regression analyses, the presence of liver metastases was associated with decreased overall survival.

In this study, we propose a high-risk profile of genetic polymorphisms related to the drug metabolism of chemotherapy in colon cancer. First, we found that *DPYD rs1801265* T/C and C/C genotypes are associated with poor prognosis. This finding aligns with the relationship between the C allele and heightened DPD enzyme activity (exonic SNP impacting DPD function), leading to increased elimination of 5-fluorouracil and reduced antitumor efficacy and the subsequent high elimination of 5-fluorouracil and low antitumor activity. The impact of DPD deficiency on toxicity is well documented [17], as are the effects on 5-FU metabolism [17,19]. However, the effect of DPD deficiency on efficacy outcomes is controversial [17,18,19]. In the TCGA analysis, we found that *DPYD* low expression is related to better overall survival compared to *DPYD* normal expression. Second, we found that the *ABCB1 rs1045642* C/C genotype is associated with a poor prognosis. The effect could be explained by the fact that these polymorphisms cause an increase in glycoprotein P (PgP) expression with the increase in efflux of 5-fluorouracil from tumor cells [20]. Third, *MTHFR rs180113* was a risk factor associated with a poor prognosis. This result is consistent with previous studies that associated the C/C genotype with low enzymatic activity, the subsequent low restitution of tetrahydrofolate, and the antitumor effect of 5-fluorouracil on *TYMS*.

Previous studies have shown that 3′UTR polymorphisms (6 bp deletion) in *TYMS* lead to destabilization of mRNA, reducing translation and TS activity. On the other hand, 3′UTR with the insertion of 6 bp leads to stability of mRNA, increasing the *TYMS* transcription/activity and the poor clinical response [3]. However, other studies have shown that 3′UTR polymorphisms predict longer disease progression and overall survival [50]. In the TCGA cohort, we found that patients with a high expression of the *TYMS*, *TK1*, *TYMP*, and *FOX1* genes are associated with longer overall survival, according to previous reports [51]. *FOXM1* plays a key role in the overexpression of genes implicated in the tumoral resistance to 5-fluorouracil treatments (*DPYD*, *TYMS*, *ABCB1*, *XRCC1*, among others) [52]. Probably, additional studies are necessary to confirm the effect of *TYMS* polymorphism and the combination or inclusion of the high-risk profile proposed here.

Our findings show that *EGFR*, *KRAS*, *NRAS*, and *PI3KCA* are not predictive factors of overall survival, neither in univariate nor multivariate analyses. These findings are consistent with previous studies showing controversial associations between *KRAS* and clinical outcomes [53,54]. Previous studies have shown a small or absent effect of *BRAF* on the prognosis of colon cancer treated with 5-fluoruracil-based chemotherapy [53,55]. However, our observations suggest that the combination of genetic polymorphism and a BRAF wild-type profile is linked to a higher risk category, leading to a less favorable prognosis among this group of Chilean patients. Specifically, these genetic traits, involving BRAF wild-type and drug metabolism polymorphisms, seem to contribute through various mechanisms to a poorer prognosis, impacting oncogenic pathways. This observation is complementary to the results obtained from the genomic drug sensitivity of cancer (GDSC) analysis. The *BRAF*-mutated status of cell lines exhibited a consistent correlation with heightened sensitivity to 5-fluorouracil, aligning with prior in vitro and in vivo xenograft model findings [55]. This effect was observed to coincide with the downregulation of *Bcl-xl* expression and the activation of the caspase-3/9 pathway [56]. Despite the small sample size and the reference studies, the *BRAF* status could be considered a predictive biomarker of 5-fluorouracil treatment in colorectal cancer. Lastly, the exploration of other genes implicated in the prognosis of colorectal cancer and their interplay with gene-drug polymorphisms, such as the *TP53* and *SMAD4* genes [56], remains a promising avenue for future research.

The primary objective of this study was to examine host characteristics, including germline polymorphisms in drug metabolism genes, and tumor characteristics, such as mutational profiles. Following the comprehensive analysis, we found that the liver metastasis status and the high-risk profile of drug-metabolism polymorphisms were associated with a poor prognosis (as indicated in Table 5) in the multivariate analysis. The effect of *BRAF* wild-type is complementary to the high-risk profile proposed. While the limited sample size significantly constrains this study, particularly in the context of multivariate regression, the initial findings underscore the necessity of expanding the sample size. In the best scenario, the cohort should have no missing data; however, this is a preliminary report from an ongoing observation study. This sample size enlargement is vital for accurately validating the influence of these biomarkers on the prognosis of colorectal cancer undergoing 5-fluorouracil-based chemotherapy. Based on the described results, patients exhibiting a high-risk profile due to genetic polymorphisms might benefit from intensified treatment, such as increased doses, more treatment cycles, or the addition of targeted therapies. However, these observations require validation in external cohorts with larger sample sizes.

## 5. Conclusions

The genetic polymorphisms *DPYD* *rs1801265*, *ABCB1* *rs1045642*, and *MTHFR* *rs180113* may serve as useful biomarkers (High-risk profile) of poor prognosis independently of *EGFR* pathway mutations in patients undergoing 5-fluorouracil chemotherapy.

## Figures and Tables

**Table 1:** General characteristics of patients.

Variable	Frequency
Gender	
Female	32 (50.8%)
Male	31 (49.2%)
Age	
Mean (SD)	63.3 (12.4)
Median [Min, Max]	66.4 [30.4, 81.8]
Histology	
Adenocarcinoma	57 (90.5%)
Adenocarcinoma Mucinous	6 (9.5%)
Localization	
Left	46 (73.0%)
Right	15 (17.5%)
N.D. *	2 (3.2%)
Liver metastases	
Yes	46 (73.0%)
No	14 (22.2%)
N.D. *	3 (4.8%)
Colectomy	
Yes	56 (88.9%)
No	7 (11.1%)
Metastasectomy	
Yes	27 (42.9%)
No	21 (33.3%)
N.D. *	15 (23.8%)
Radiotherapy	
Yes	11 (17.5%)
No	52 (82.5%)
Monoclonal antibodies therapy	
Yes	14 (22.2%)
No	49 (77.8%)
First line of treatment	
CapeOx	26 (41.3%)
Folfox	32 (50.8%)
Folfiri	5 (7.9)
Second line of treatment (FOLFOX or FOLFIRI)	
Yes	37 (56.8%)
No	26 (41.3%)

* N.D. = No data.

**Table 2:** Genotype frequencies of patients.

Variable	Frequency
*TYMS 3′UTR 6bp ins-del (rs151264360)*	
DEL/DEL	31 (49.2%)
INS/DEL	32 (50.8%)
*GSTP1 c.313A>G (rs1695)*	
A/A	20 (31.7%)
G/A	28 (44.4%)
G/G	15 (23.8%)
*DPYD* c.1905+1 G>A (DPYD*2) (rs3918290)	
G/G	63 (100%)
G/A	0 (0%)
A/A	0 (0%)
*c.2846A>T (rs67376798)*	
A/A	1 (1.6%)
T/A	1 (1.6%)
T/T	61 (96.8%)
c.1679T>G (DPYD*13) (rs55886062)	
T/T	63 (100%)
T/G	0 (0%)
G/G	0 (0%)
c.85T>C (DPYD*9) (rs1801265)	
T/T	37 (58.7%)
C/T	19 (30.2%)
C/C	6 (9.5%)
N.D.	1 (1.6%)
*ABCB1* *c.3435C>T (rs1045642)*	
T/T	9 (14.3%)
C/T	31 (49.2%)
C/C	21 (33.3%)
N.D.	2 (3.2%)
*c.1236 T>C (rs1128503)*	
T/T	6 (9.5%)
C/T	41 (65.1%)
C/C	15 (23.8%)
N.D	1 (1.6%)
*ABCC2 c.-24C>T (rs717620)*	
C/C	46 (73.0%)
C/T	11 (17.5%)
T/T	2 (3.2%)
N.D.	4 (6.3%)
*MTHFR c.1409A>C (rs1801131)*	
A/A	33 (52.4%)
A/C	21 (33.3%)
C/C	8 (12.7%)
N.D.	1 (1.6%)

N.D. = No data (due to sample shortage).

**Table 3:** Molecular somatic profiles of patients.

Tumor Mutation	Frequency
*BRAF*	
Mutated (*V600E*)	7 (11.1%)
Wild-type	58 (88.9%)
KRAS mutations *	
Mutated	22 (34.9%)
Wild-type	41 (65.1%)
NRAS mutations **	
Mutated	7 (11.1%)
Wild-type	56 (88.9%)
PI3KCA mutations ***	
Mutated	7 (11.1%)
Wild-type	56 (88.9%)
*AKT1 E17K*	
Mutated	2 (3.2%)
Wild-type	61 (96.8%)

* KRAS 1213, KRAS117, KRAS61, KRAS146, and KRAS59 (Entrogen Colorectal Cancer Mutation Detection Panel). ** NRAS1213, NRAS117, NRAS61, NRAS146, and NRAS59 (Entrogen Colorectal Cancer Mutation Detection Panel). *** PI3KCA542545 and PI3KCA1047 (Entrogen Colorectal Cancer Mutation Detection Panel).

**Table 4:** Univariate analysis *.

Variables	Patients (*n*)	HR	95% CI Lower-Upper	*p*-Value **
Liver metastases				
Yes	46	3.51	1.52–8.07	**0.003**
No	14	Ref.		
Colectomy				
Yes	56	0.480	0.214–1.08	0.079
No	7	Ref.		
*GSTP1 rs1695*				
Yes (G/G)	15	0.484	0.234–1.00	0.05
No (G/A+A/A)	48	Ref.		
*DPYD rs1801265*				
Yes (C/C + C/T)	25	1.819	1.03–3.19	**0.0377**
No (T/T)	37	Ref.		
*ABCB1 rs1045642*				
Yes C/C	21	1.782	1.00–3.16	**0.0483**
No (C/T+T/T)	40	Ref.		
*MTHFR rs180113*				
Yes C/C	6	2.295	1.05–4.97	**0.0352**
No (A/C+A/A)	54	Ref.		
*TYMS rs151264360*				
Yes Ins/Del	32	2.169	1.21–3.86	**0.0087**
No Del/Del	31	Ref.		
Mutated *PI3KCA*				
Yes	7	0.271	0.084–0.876	**0.0292**
No	56	Ref.		
*BRAF*				
Wild-type	56	0.28	0.087–0.909	**0.034**
Mutated (*V600E*)	7	Ref.		
High-Risk Profile ***				
Yes	41	2.06	1.13–3.74	**0.018**
No	21	Ref.		
High-Risk Profile *** + BRAF wild-type patients				
Yes	38	2.80	1.55–5.06	**<0.005**
No	25	Ref.		

* Only associations with a *p*-value < 0.1 are shown and selected for multivariate analysis. ** *p* < 0.05 is statistically significant (in bold). *** The risk genotype profile includes *DPYD rs1801265* T/C + C/C genotypes, *ABCB1 rs1045642* C/C genotype, and *MTHFR rs180113* C/C genotype. HR = Hazard Ratio; CI = 95% Confidence Interval (Cox regression). Ref = Reference Category.

**Table 5:** Multivariate analysis (final model, *n* = 58 subjects).

Variables	Patients (*n*)	HR	95% CI Lower-Upper	*p*-Value *
Liver metastases				
Yes	45	3.69	1.49–9.09	**0.004**
No	13	Ref.		
*DPYD* *rs1801265*				
Yes (C/C + C/T)	23	1.88	0.99–3.54	0.052
No (T/T)	35			
*ABCB1 rs1045642*				
Yes C/C	20	2.62	1.37–4.99	**0.003**
No C/T + T/T	38	Ref.		
*MTHFR rs180113*				
Yes C/C	8	2.63	1.13–6.15	**0.004**
No A/C + A/A	50	Ref.		

* *p* < 0.05 is statistically significant (in bold). Concordance of the model (C) = 0.692 HR = Hazard Ratio; CI = 95% Confidence Interval (Cox regression). Ref = Reference Category.

**Table 6:** Multivariate analysis (association between high-risk profile and mutational status) (*n* = 58 subjects).

a. High-Risk Profile * and *BRAF V600E* Mutation.				
Variables	Patients (*n*)	HR	95% CI Lower-Upper	*p*-Value **
High-risk presence				
Yes	38	2.18	1.15–4.11	**0.017**
No	20	Ref.		
Liver metastases				
Yes	45	3.34	1.39–8.05	**0.006**
No	13	Ref		
*BRAF V600E*				
Mutated	7	0.41	0.12–1.39	0.153
Wild-type	51	Ref.		
**b. High-risk profile** * and *KRAS* mutation.				
**Variables**	**Patients (*n*)**	**HR**	**95% CI** **Lower-Upper**	*p*-value **
High-risk presence				
Yes	38	2.28	1.20–4.33	**0.012**
No	20	Ref.		
Liver metastases				
Yes	45	4.71	1.91–11.6	**<0.005**
No	13	Ref		
*KRAS*				
Mutated	21	0.59	0.31–1.11	0.105
Wild-type	37	Ref.		
**c. High-risk profile** * and *PI3KCA* mutation.				
**Variables**	**Patients (*n*)**	**HR**	**95% CI** **Lower-Upper**	*p*-value **
High-risk presence				
Yes	38	2.43	1.26–4.66	**0.007**
No	20	Ref.		
Liver metastases				
Yes	45	4.08	1.68–9.86	**0.002**
No	13	Ref		
*PI3KCA*				
Mutated	6	0.22	0.05–0.95	**0.042**
Wild-type	52	Ref.		

* The risk genotype profile includes *DPYD* *rs1801265* T/C + C/C genotypes, *ABCB1* *rs1045642* C/C genotype, and *MTHFR* *rs180113* C/C genotype. ** *p* < 0.05 is statistically significant (in bold). HR = Hazard Ratio; CI = Confidence Interval (Cox regression).

**Table 7:** Clinical response according to high-risk *BRAF* wild-type patients *versus* all patients (*n* = 58 subjects).

Variables	Patients (*n*)	HR	95% CI Lower-Upper	*p*-Value *
High-risk ** + *BRAF* wild-type patients				
Yes	35	2.71	1.46–5.01	**0.001**
No	23	Ref.		
**Liver metastases**				
Yes	45	3.55	1.52–8.29	**0.003**
No	13	Ref		

* *p* < 0.05 is statistically significant (in bold). ** The risk genotype profile includes *DPYD* *rs1801265* T/C + C/C genotypes, *ABCB1* *rs1045642* C/C genotype, and *MTHFR* *rs180113* C/C genotype. HR = Hazard Ratio; CI = Confidence Interval (Cox regression).

## Data Availability

The datasets generated for this study are available on request to the corresponding author.

## Supplementary Materials

The following supporting information can be downloaded at: https://www.mdpi.com/article/10.3390/curroncol31010018/s1. It includes Table S1, which details the measurements and variables utilized in this study.
