# Supplementary material for: Genetic Polymorphisms and Tumoral Mutational Profiles over Survival in Advanced Colorectal Cancer Patients: An Exploratory Study
Source: Curr Oncol. 2024 Jan 3;31(1):274–95. doi: 10.3390/curroncol31010018 (PMC10814806; doi:10.3390/curroncol31010018)
Supplement: Supplementary file 1 [file curroncol-31-00018-s001.zip › curroncol-2716906-supplementary.pdf]

Supplementary material

Supplementary material

Table S1. Variables used in this study.

**Variable**	**Description (groups or units)**
Gender	The gender of the patients (female or male)
Age	The age of patients at diagnosis (years)
Histology	Histological type of colon cancer (adenocarcinoma or mucinous adenocarcinoma)
Localization	Localization of lesions in the colon (left or right)
Liver metastases	Presence or absence of liver metastases
Colectomy	Presence or absence of colectomy
Metastasectomy	Presence or absence of metastectomy
Radiotherapy	Presence or absence of radiotherapy to colon cancer
Monoclonal antibodies therapy	Use of monoclonal antibodies therapy to colon cancer (e.g CUALES?)
Second line of treatment	Type of second life of treatment: FOLFOX or FOLFIRI
Overall survival	Survival from diagnosis until death for any cause

Note: All patients received FOLFOX or CapeOx as the first-line treatment.

YO CREO QUE LO QUE PEDÍA EL REVISOR ERA ALGO UN POCO MÁS COMPLETO
